# An expanded role for single-cell chemical genomics profiling in drug discovery

**DOI:** 10.1042/BCJ20253273

**Published:** 2026-01-21

**Authors:** Adeya Wyatt, Kevin Hoffer-Hawlik, Ross M. Giglio, Elham Azizi, José L. McFaline-Figueroa

**Affiliations:** 1 Department of Biomedical Engineering, Columbia University, New York, NY 10027, U.S.A.; 2 Irving Institute for Cancer Dynamics, Columbia University, New York, NY 10027, U.S.A.; 3 Department of Molecular Pharmacology and Therapeutics, Columbia University Medical Center, New York, NY 10032, U.S.A.; 4 Department of Computer Science, Columbia University, New York, NY 10027, U.S.A.; 5 Herbert Irving Comprehensive Cancer Center, Columbia University, New York, NY 10032, U.S.A.; 6 Data Science Institute, Columbia University, New York, NY 10027, U.S.A.

**Keywords:** CRISPR, drug discovery and design, functional genomics, high-throughput screening, pharmacogenomics

## Abstract

The integration of single-cell genomics into the chemical genetics paradigm is reshaping how researchers profile drug activity, prioritize lead candidates, and uncover new therapeutic opportunities. Traditional chemical genetic approaches, though instrumental in linking compounds to cellular phenotypes, often rely on bulk measurements that obscure important cellular heterogeneity and limit insight into mechanisms of action. By contrast, single-cell technologies offer a transformative view of how compounds influence diverse cell types and states, capturing nuanced molecular responses that further our understanding of efficacy, resistance, and polypharmacology. From cancer to neurodegenerative disorders and other disease contexts, single-cell chemical profiling enables a more precise annotation of drug-induced effects, revealing differential responses across cellular subpopulations. These methods help identify both beneficial and adverse outcomes that may not be readily predicted by a compound’s structure or known targets, enhancing preclinical prioritization and supporting rational drug repurposing strategies. As these technologies mature, advances in multiplexing, multimodal profiling, and computational analysis are expanding their scalability and applicability to increasingly complex models. The resulting data-rich assays are poised to bridge critical gaps between compound screening and clinical relevance. This review highlights the evolution of chemical genomics toward single-cell resolution and outlines emerging opportunities to leverage these methods throughout the drug discovery pipeline, from early preclinical prioritization to late-stage repurposing, ultimately accelerating the development of safer, more effective therapies.

## Introduction

The advent of genomic technologies has significantly enhanced our ability to catalog how perturbations influence cellular states [1–6]. In the context of disease, genomics tools can help identify actionable disease drivers [7–9], map genetic variation to cellular phenotypes [10–13], and define regulatory networks associated with therapeutic efficacy [14–16]. More recent advances applying these technologies at single-cell and spatial resolution have further refined our understanding of disease, pinpointing specific cell types and states underlying disease phenotypes [17] and highlighting a basis for heterogeneity in disease manifestation [18].

Chemical genetics revolutionized our ability to dissect how thousands of compounds and biologics alter gene function and cellular phenotypes [19]. High-throughput chemical genetic screens applied across genetically diverse cellular models, both naturally occurring [2] and engineered through genetic manipulation [20], have helped define the genetic dependencies of cellular responses to various exposures. Many genetic screens are limited to gross phenotypic (such as viability and proliferation) or highly specific molecular readouts (such as transcriptional reporters and enzymatic activity). While valuable, these approaches often limit insight into mechanisms of action or off-target effects. Over the past two decades, the integration of large-scale genomic profiling into chemical genetics has offered a more comprehensive understanding of cellular responses to perturbations and laid the foundation for the extension of this field into chemical genomics [21].

Drug discovery strategies are broadly categorized into target-based drug discovery (TDD) and phenotypic drug discovery (PDD) [22]. In TDD, a molecular target is nominated a priori based on biological or genetic evidence, and screening efforts focus on identifying compounds or biologics optimized for their ability to modulate that target. The TDD approach is analogous to reverse genetic screens in which perturbation of a defined gene is used to interrogate downstream phenotypes [19]. In PDD, compounds are prioritized based on their ability to elicit a cellular phenotype of interest, without requiring prior knowledge of the underlying target. This approach parallels forward genetic screens, which begin from a phenotype and work backward to identify causal genes. In the context of chemical genomics, target-based campaigns use high-throughput, information-rich profiling and computational tools to understand how modulation of a target reshapes biological states [23]. In phenotypic screens, it enables the deconvolution of targets and pathways underlying active compounds by linking exposure to detailed molecular response profiles [24].

In drug discovery, whether in target-based or phenotypic campaigns, there is an inherent balance between employing well-established, low-information-content assays that are specific, rapid, and scalable [25] and leveraging unbiased, information-rich profiling [24]. However, drug discovery efforts may lack a comprehensive annotation of the diverse molecular effects induced by a compound. This gap can result in unanticipated drug-induced effects, an incomplete understanding of a drug’s mechanism of action, and poor clinical translation [26–28]. By systematically integrating large-scale profiling of drug-induced molecular effects alongside standard targeted assays that define on-target activity, researchers can improve the characterization of a drug’s mechanism of action and its often complex (poly-)pharmacology.

Within the drug development pipeline, chemical genomics tools present a unique opportunity to enhance the characterization of compound libraries, promising lead candidates, and both investigational and approved drugs based on their ability to induce distinct molecular states. Applying this paradigm early in the pipeline would enable the identification of beneficial and detrimental effects that may not be apparent from a compound’s chemical structure, class, or annotated mechanism of action. Such insights have the potential to improve prioritization, mitigate unforeseen negative effects, and refine therapeutic strategies.

**Figure 1:**
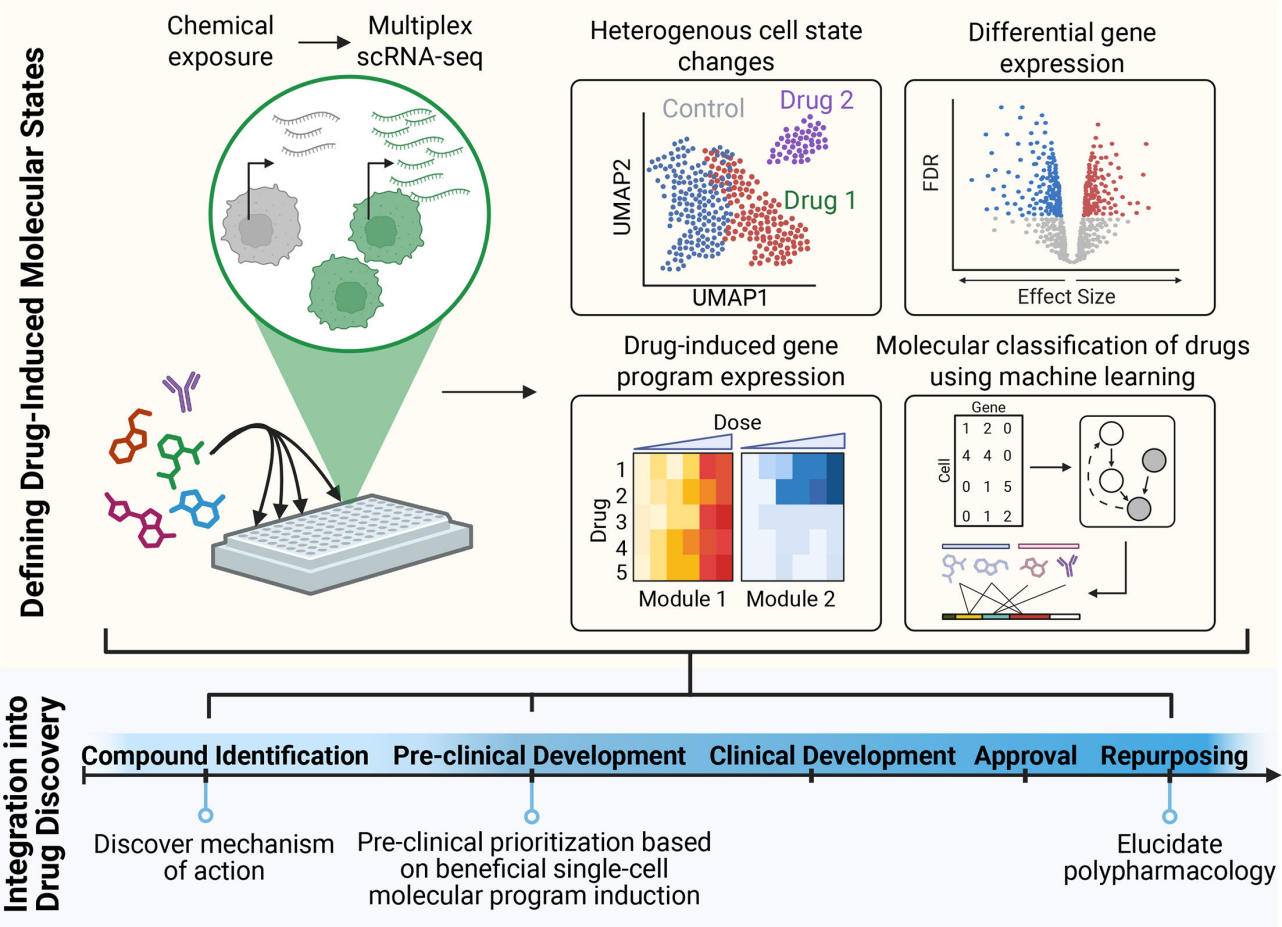
High-throughput single-cell chemical genomics. Results can drive progress in compound identification, preclinical prioritization, and the characterization of a drug’s polypharmacology, as well as facilitate drug repurposing efforts.

In this review, we explore the methodological and computational advances that can be readily applied to the drug discovery pipeline. Specifically, we highlight areas where high-throughput single-cell chemical genomics profiling can drive progress in compound identification, preclinical prioritization, and the characterization of a drug’s polypharmacology, as well as facilitate drug repurposing efforts (Figure 1). For a more detailed discussion on target identification, disease-specific drug discovery, genomics applications, quality control, and best practices, we refer readers to the following reviews [29–33].

### Population-averaged chemical genomics approaches

Before the rapid advancement of next-generation sequencing (NGS), high-throughput parallel measures of gene expression first took place on DNA microarrays [34], capturing relative mRNA levels using hybridization to gene-specific probes. Marton et al. first established the idea of a chemical ‘signature,’ or a gene expression footprint, demonstrating not only on-target calcineurin signaling inhibition-induced expression signatures in *Saccharomyces cerevisiae* but also off-target *GCN4*-regulated transcriptional responses [3]. Later, Hughes et al. built on this with the idea of collecting a ‘compendium’ of signatures, showing in *S. cerevisiae* that deletions in genes involved in similar pathways result in more similar signatures compared with gene deletions involved in separate pathways [35]. This idea proved its value as the authors assigned function to several unannotated yeast ergosterol pathway genes; their search narrowed substantially simply by inspecting how a signature for a gene of unknown function clustered with those of known genes, and the authors suggest the future use of the approach for chemical perturbations [35].

The compendium approach was applied in what would become the Connectivity Map (CMap), part of the National Institutes of Health (NIH) Common Fund’s Library of Integrated Network-based Cellular Signatures (LINCS) program [36]. Originally a collection of 453 perturbation-induced expression signatures across four human cancer cell lines measured by DNA microarrays [37], the subsequent CMap studies measure perturbation response by L1000, an assay using bead hybridization and fluorescent measurements of 978 landmark transcripts as a proxy for the expression of the transcriptome [2]. The approach has allowed for the profiling of a large number of perturbations with the latest iteration containing 1.16M replicate-collapsed signatures from >80 k perturbations (33,609 compounds; 9288 genes) across >200 cell models [38]. By connecting signatures of uncharacterized chemicals to those of known chemicals and genetic perturbation via gene loss-of-function, CMap authors classified previously unknown ROCK and MTOR/PI3K inhibitors and discovered the first inhibitor against CSNK1A1. Resources at this scale have the potential to enable discovery of effective therapeutics, especially when coupled with orthogonal resources like the Cancer Dependency Map [1,39,40], an encyclopedic endeavor to uncover genetic dependencies across an ever-growing collection of pan-cancer cell lines, including the extensive genomic characterization of these cell lines [41–43], and examining viability changes in response to a diverse set of drugs [44,45]. Notably, the L1000 assay, while advantageous for its low cost and scale, is limited by the number of genes measured, requiring the imputation of 9196 of 11,350 unmeasured genes. The authors note that missing information from the L1000 readout could contribute to the inability to connect 37% of the screened compounds to their expected target and that higher sensitivity measurements should be considered for future studies.

More recently, with the lowering costs of sequencing and transcriptome profiling, bulk RNA-seq chemical genomic approaches were developed to address the challenge of faithfully measuring the whole transcriptome while maintaining high-throughput capacity. Landmark studies, PLATE-seq [4] and later DRUG-seq [46], established multi-well plate-based bulk RNA-seq protocols to measure genome-wide expression changes in response to chemical or genetic perturbation by leveraging well-specific reverse transcription barcodes. The PLATE-seq and DRUG-seq studies went on to collect signatures from 184 and 433 chemicals, respectively, demonstrating the feasibility of collecting unbiased transcriptomic responses to study the molecular response to chemicals from a mechanistic perspective.

In addition to gene expression profiling, other groups have enabled assays with orthogonal genomic measurements of drug response, such as epigenomics and proteomics. With regard to the latter, efforts to measure protein-based drug signatures [47] have resulted in the discovery and further characterization of HDAC inhibitors [48,49], JNK inhibitors [50], adrenergic receptor antagonists [51], CDK inhibitors [52], and BRAF inhibitors [53], to name several examples, providing mechanistic insights into drug–protein interaction, intra-protein stability and disruption, and interactions within protein complexes [54].

Population-averaged, or bulk, approaches highlight the utility of collecting and comparing perturbation-induced gene expression signatures. From these data and with follow-up validation, researchers were able to assign function to uncharacterized genes, classify or reclassify chemicals by mechanism of action, and identify off-target inhibition. These previous studies laid the foundation for the application of large-scale chemical genomics and continue to be resources in forming hypotheses for drug discovery and development. Over the last two decades, advances in single-cell genomics technologies have led to the discovery of striking heterogeneity in cell type composition and an astounding diversity in cellular state across normal and pathological conditions [55–60]. Population-averaged approaches, with their limited sensitivity to detect these differences, average over heterogeneity, often leading to a muted view of induced changes and, in some instances, can reverse the true trends in the data, a phenomenon known as Simpson’s paradox [61]. Of relevance to chemical genomics, bulk averaging can mask the response of clinically relevant cellular subpopulations and ignore heterogeneity in drug-induced molecular responses. Lastly, despite atlas-scale efforts, it is nearly impossible for a single resource to characterize perturbation signatures of interest to researchers (i.e. provide data for the inhibitor of interest in a particular model). Therefore, accessible screening methods, technologies that account for heterogeneity, and computational tools capable of inferring drug-induced effects are a necessity to uncover unique genotype- and therapy-specific transcriptional responses.

### Chemical genomics at single-cell resolution

Population-averaged, bulk chemical genomics approaches coupled with high-throughput screens led to a significant advance in the ability to perform molecular characterization within the drug-discovery pipeline. However, these methods are still limited by (1) the inability to capture heterogeneous changes in cell state, which can frequently occur even within seemingly homogeneous cellular systems, and (2) the lack of resolution in complex heterogeneous systems compatible with high-throughput screening (e.g. organoids) to monitor cell type and cell state specific responses and how changes in cell type composition correlate with drug response.

In the past 15 years, single-cell omics techniques have revolutionized our ability to identify the cellular basis of disease pathology and aid in the prioritization of therapeutic approaches. Leveraging the resolution of single-cell omics technologies is quickly enabling the discovery of translationally impactful correlations between precise cell-state measurements and disease, which have the opportunity to feed back to advance drug discovery. Within the purview of single-cell chemical genomics, the systematic molecular screening of drugs at single-cell resolution has the potential to expedite the drug discovery pipeline. Similar to bulk methods, it can enable annotations that inform treatments with increased efficacy and minimize detrimental effects, uncovering mechanisms to combat drug resistance all while determining whether these properties vary across cellular subpopulations. For example, in the context of cancer, single-cell transcriptomics approaches have been applied to gain insight into the resistance continuum [62], the gradual adaptation of cancer cells to cancer therapy with increasing fitness. Single-cell profiling of a *BRCA2*-deficient high-grade serous ovarian cancer cell line with varying concentrations of treatment identified vulnerabilities to PARP inhibition with olaparib through the combinatorially targeting of glutaminase 1 with CB-839, increasing treatment efficacy compared with monotherapy PARP inhibition treatment, and inducing synthetic lethality in *BRCA1-* and *BRCA2-*deficient tumors [62]. Single-cell genomics technologies have similarly been applied to define how canonical amyloid-β and tau pathologies regulate microglia subtypes during Alzheimer disease progression through scRNA-seq [63] and sex-specific gene-expression changes in neurodegenerative diseases that can be monitored by profiling cell types of the blood [64].

**Figure 2:**
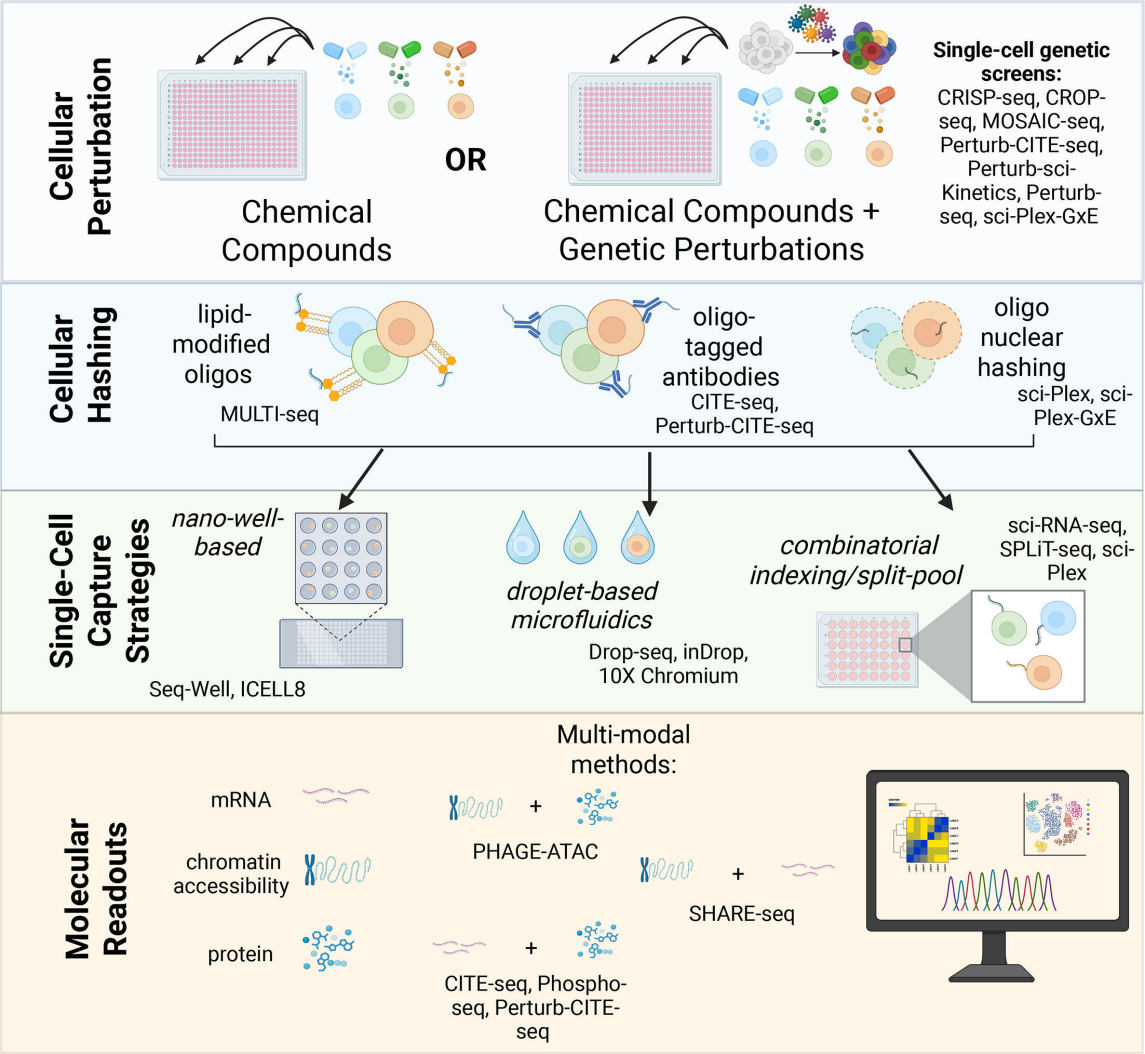
An overview of the general workflow of wet lab methods for chemical genomics screening. After exposing cells to chemical compounds, various hashing cellular and nuclear hashing methods may be used before applying one of the three single-cell genomics strategies: nano-well based, droplet-based microfluidics, or combinatorial indexing/split-pool. Finally, the high-throughput, multiplexed data produced by these techniques are computationally analyzed, with some methods enabling multi-modal insights.

The large number of conditions within a high-throughput chemical screen brings several challenges to the use of single-cell molecular readouts, in particular, the need for technologies with sufficient throughput and the need to minimize technical variation across batches. Thresholds for efficiency and cost-effectiveness should also be considered when choosing an appropriate readout. Currently, there are various single-cell genomics approaches that have or can be readily adapted to chemical genomic profiling (nanowell, droplet-based microfluidic, and combinatorial indexing/split-pool techniques), each with differing characteristics in sensitivity, cost, and throughput. We first discuss the general characteristics of methods that can be applied to routinely capture at least thousands of cells per experiment, focusing on the measure of cellular transcriptomes and how they are suitable for single-cell chemical genomics profiling, followed by introducing a variety of multiplexing strategies at the exposure or cellular level that can minimize technical noise when applied to large screens and can be leveraged to increase the throughput of these techniques. Lastly, we comment on how further innovation on these platforms has enabled multi-modal profiling approaches at the level of the proteome, epigenome, and genome (Figure 2).

Nano-well-based strategies (e.g. Seq-Well [65] and ICELL8 [66]) use gravity (sedimentation) to physically isolate individual cells into nanowell arrays, resulting in an efficient, simple, low-cost system. Despite having a lower overall throughput than the droplet-based microfluidic and combinatorial indexing/split-pool techniques described below, nanowell-based methods have a high capture rate for a cell’s transcriptome and are ideal for low cell number samples [67]. The scalability and native throughput of nanowell-based methods, however, are limited by the number of array wells, although cellular hashing techniques, described later, could allow for array ‘superloading’ to increase throughput in single-cell chemical genomics screens.

Droplet-based microfluidic techniques are the most commonly used single-cell methods for high-throughput single-cell scRNA-seq. In this approach, individual cells and barcoded beads are co-encapsulated within an aqueous/oil emulsion within nanoliter droplets that uniquely tag a cellular transcriptome. Drop-seq [68] and inDrop [69] constitute the first iterations of these methods. Drop-seq, developed by Macosko and colleagues, separates single cells into droplets, capturing single-cell transcriptomes attached to microparticles (STAMPs) for reverse transcription, amplification, and sequencing using the STAMP barcodes to identify the cell of origin. InDrop (indexing droplets) encapsulates cells with a reverse transcription mix and hydrogel beads containing primers released by UV irradiation. Prior to sequencing, this method employs T7 RNA polymerase for linear amplification via *in vitro* transcription. The commercialization of droplet-microfluidic approaches (e.g. the Chromium system from 10X Genomics) led to an exponential increase in the accessibility of researchers to single-cell RNA-seq technology and the innovation of novel assays on the platform. The throughput of commercial droplet-based microfluidic techniques allows for the capture of up to 10–20,000 cells per unique sample. Cellular hashing approaches, described below, can be used to increase this throughput by overloading nanoliter droplets. However, these approaches have limits on the size of cells that can be captured (large cells may block the device) and can include technical noise and background associated with ambient RNA [65].

Combinatorial indexing or split/pool methods (e.g. sci-RNA-seq [70] and SPLiT-seq [71]) constitute highly scalable single-cell genomics technologies. In contrast to other methods, cells are not physically isolated for library preparation, but rather single cells or nuclei are uniquely labeled via a combination of barcodes across several indexing rounds. Throughput scales rapidly with the number of indexing rounds allowing for the profiling of millions of cells within one experiment. The approaches have allowed for comprehensive profiling of millions of cells representing cell types and cell states across multiple organisms [72,73]. Similar to microfluidic approaches, a key consideration is that the number of available cellular barcodes is significantly larger than the number of cells, minimizing barcode collisions [74]. In the context of drug discovery, this level of scale pairs well with the notion of large throughput screens and was demonstrated in our development of the sci-Plex pipeline for multiplex single-cell chemical transcriptomics [5]. Recently, the suitability of these technologies for large-scale single-cell chemical genomics profiling was excitingly highlighted by the release of the Tahoe-100M, a 100 million cell dataset, collected using a split-pool technique, of mosaic collections of cancer cell lines exposed to hundreds of compounds at various drug/dose combinations [75].

Drug-induced cellular states can be characterized by changes in RNA transcription, chromatin accessibility, protein expression, metabolisms, or other molecular changes. Any one technology (genomics, transcriptomics, proteomics, and metabolomics) fails to capture the full scope of a single cell’s identity, making multi-modal multiplexed methods ideal for deeper insights into cellular drug response. Multiple technologies have been developed to measure additional aspects of the central dogma at single-cell resolution, frequently in combination with other measurements [ [76–82]]. For example, CITE-seq and Phospho-seq profile both the transcriptome and quantify surface or intracellular proteins with oligonucleotide-labeled antibodies [83,84]. Assays to profile chromatin accessibility profiling, such as the assay for transposase accessible chromatin (ATAC-seq [85]) have been extended to droplet-based microfluidics [86] and combinatorial indexing [87] approaches for gene expression analysis. PHAGE-ATAC measures phage-based multiplex protein measurements and chromatin accessibility through scATAC-seq in parallel, utilizing phages for epitope profiling and phage libraries to select antigen-specific libraries [88]. SHARE-seq and sci-CAR simultaneously measure chromatin accessibility and gene expression, linking accessibility to transcription across cell states [89,90]. DEFND-seq co-profiles mRNA and genomic DNA (gDNA) from an individual cell’s nucleus, building on ATAC-seq by revealing nucleosome positioning [91]. NEAT-seq [92] reduces non-specific staining of barcoded antibodies to nuclear proteins by conjugating *Escherichia coli* ssDNA binding protein, enabling simultaneous profiling of intra-nuclear proteins, chromatin accessibility, and gene expression. The epigenome has also been studied at single-cell resolution using DNA methylation profiling via bisulfite conversion with high-throughput single-cell combinatorial indexed techniques using sciMETv2 [93], where non-methylated cytosine bases are converted into uracil and sequenced as thymine. Lastly, techniques for whole genome sequencing at single-cell resolution (sc-WGS) and the direct capture of primary DNA sequence to profile mutations and areas of genetic variation (e.g. SMOOTH-seq [94]) provide the final piece of the central dogma. Exciting next steps for the fields include the incorporation of metabolic measurements to arrive at comprehensive measures of cellular state. Single-cell metabolic regulome profiling (scMEP) [95], for example, an antibody-based, single-cell metabolic profiling method, has been used to define the metabolic states of human cytotoxic T cells. While techniques that simultaneously assess the genome, transcriptome, and proteome of an individual cell together have not been widely established, several techniques do provide multi-modal insight that leads to more detailed cell profiling, better potential drug-target identification, and broader drug response prediction. Systems such as Element Bioscience’s AVITI24 are working to close this gap by combining NGS, RNA capture, proteomics, and morphology of a sample in one platform [96].

Cellular hashing approaches for single-cell genomics allow multiple samples, such as cellular models exposed to diverse chemical libraries, to be pooled and processed jointly within one experiment, increasing throughput, minimizing batch effects, and reducing costs [97]. Most approaches involve the addition of unique DNA-barcoded tags assigned to each sample and synthetic analytes to be captured within each methodology. These tags enable pooled samples to be deconvolved in downstream analysis and individual cells to be matched to specific conditions. A range of cellular hashing methods has been optimized and implemented. One of the first examples of cellular hashing was developed in 2018 by Stoeckius et al. as a modification of the multiplexed single-cell multi-modal measurement technique CITE-seq [98]. This multiplexing strategy allows for the increase of parallel experiments and helps identify cell multiplets signaling by using a defined set of oligo-tagged antibodies to attach to proteins on the cell surface. MULTI-seq [99], a technique that specifically tags cells with lipids and cholesterol-conjugated oligonucleotides to barcode live cells and nuclei. Using MULTI-seq, McGinnis et al. identified transcriptional responses of myoepithelial and luminal epithelial cells across co-culture conditions and after induction of transforming growth factor beta (TGF-β) signaling. The combinatorial indexing-based sci-Plex approach, taking advantage of the inherent affinity of DNA molecules to the cell nucleus, involves attaching single-stranded DNA oligonucleotide barcodes to permeabilized cells. This low-cost multiplexing strategy coupled to combinatorial indexing sci-RNA-seq3 was used in an unbiased single-cell chemical genomics screen profiling thousands of unique combinations of cell model, drug, and dose, identifying a novel metabolic basis of the molecular response of cells to histone deacetylase inhibition [5] and classifying related classes of small molecule inhibitors (EGFR inhibitors) by their ability to induce distinct molecular programs [100].

The tracking of genomic heterogeneity within captured cellular transcripts also allows for multiplexing across diverse cellular models. Demuxlet [101] and Mix-seq [102] use sample-specific demultiplexing based on genetic single-nucleotide polymorphism to deconvolve mixtures of cell types. Mix-seq demonstrates the ability to use this approach to multiplex conditions across diverse cell models exposed to differing drugs and multiple time points [102]. Lastly, the incorporation of heritable barcodes amenable to capture via single-cell genomics techniques can be used for multiplexing and to track the evolution of cells under therapy. CellTag Indexing [103] tracks cells and cellular lineages across different conditions over different time points by the expression of randomized molecular indexes within RNA polymerase II driven transcripts via sequential lentiviral delivery and has recently been extended to multimodal profiling [104]. Cheng and colleagues recently used CellTag to generate a lentivirus barcode library and observed an association between the induction of resistance to targeting of the kinesin motor KIF11 by ispinesib in glioblastoma (GBM) cells and a transition to the improved-survival-associated proneural glioma subtype [105].

The addition of single-cell genetic screens within the context of the chemical genomic pipelines allows for the ability to systematically dissect which genes are causative for drug-induced molecular changes. Beginning in 2016, the development of the Perturb-seq [106,107], CRISP-seq [108], CROP-seq [109], and MOSAIC-seq [110] approaches, which couple CRISPR-based genetic perturbations with a single-cell transcriptomics readout, revolutionized our ability to determine how gene activity alters gene expression networks. The first iteration of these methods relied on capture of CRISPR single guide RNA (sgRNA) identity by capturing a polyadenylated transcript containing a distal barcode that reports on the delivered sgRNA or embedding of the sgRNA within the 3′ untranslated region (UTR) of a reporter transcript in droplet-microfluidics based single-cell RNA-seq. Distal barcode methods can suffer from decreased statistical power due to lentiviral recombination during pooled lentivirus generation [111], which can be minimized by creating viral particles individually and pooling for transduction [107]. More recently, innovations have allowed for direct capture of short sgRNAs [112,113], which fully mitigates challenges associated with distal barcodes. Recently, the sci-Plex-GxE [114] and Perturb-sci-Kinetics [115] frameworks have demonstrated the ability to couple single-cell genetic screens with high-throughput combinatorial indexing RNA-seq, applying them to identify the regulation of resistance programs downstream of drug-induced transcription and the regulation of RNA synthesis, respectively.

Advancements in the scalability of high-throughput screens, multiplexing strategies, and cellular hashing have led to the generation of large, complex datasets. Despite the fact that data are becoming increasingly multiplexed as the scope of single-cell analysis increases, the single-cell chemical genomics field is limited by both technique efficiency and older demultiplexing analysis. To truly make use of single-cell chemical genomics and gain biological insight to be applied to drug discovery, innovation is still needed to produce more robust readouts while still facilitating scale. Newer computational techniques are also needed in order to extract meaningful information from these sparse, large-scale screens regarding cellular response to drug exposure. To elucidate more mechanisms of action, better dissect immune response, and unbiasedly catalog drugs with single-cell chemical genomics, more advancements must be made in computational frameworks to analyze increasingly complex, high-throughput single-cell data.

### Computational methods to gain insight from highly multiplex chemical genomics screens

The applications of large-scale chemical genomics screens to drug discovery require the development of novel computational tools that account for considerations specific to multiplex chemical screens and allow us to gain insight from these extraordinarily high-dimensional datasets while accounting for confounding technical artifacts of single-cell experiments. As discussed in [29], the adoption of high-throughput drug screens with molecular readouts has already revolutionized drug discovery, allowing for the identification of therapeutic cellular targets and a better understanding of drug mechanism of action. Further, these assays are well poised to generate the necessary data toward the rational design of drug combinations with predicted therapeutic effects and to inform drug repurposing efforts.

**Figure 3:**
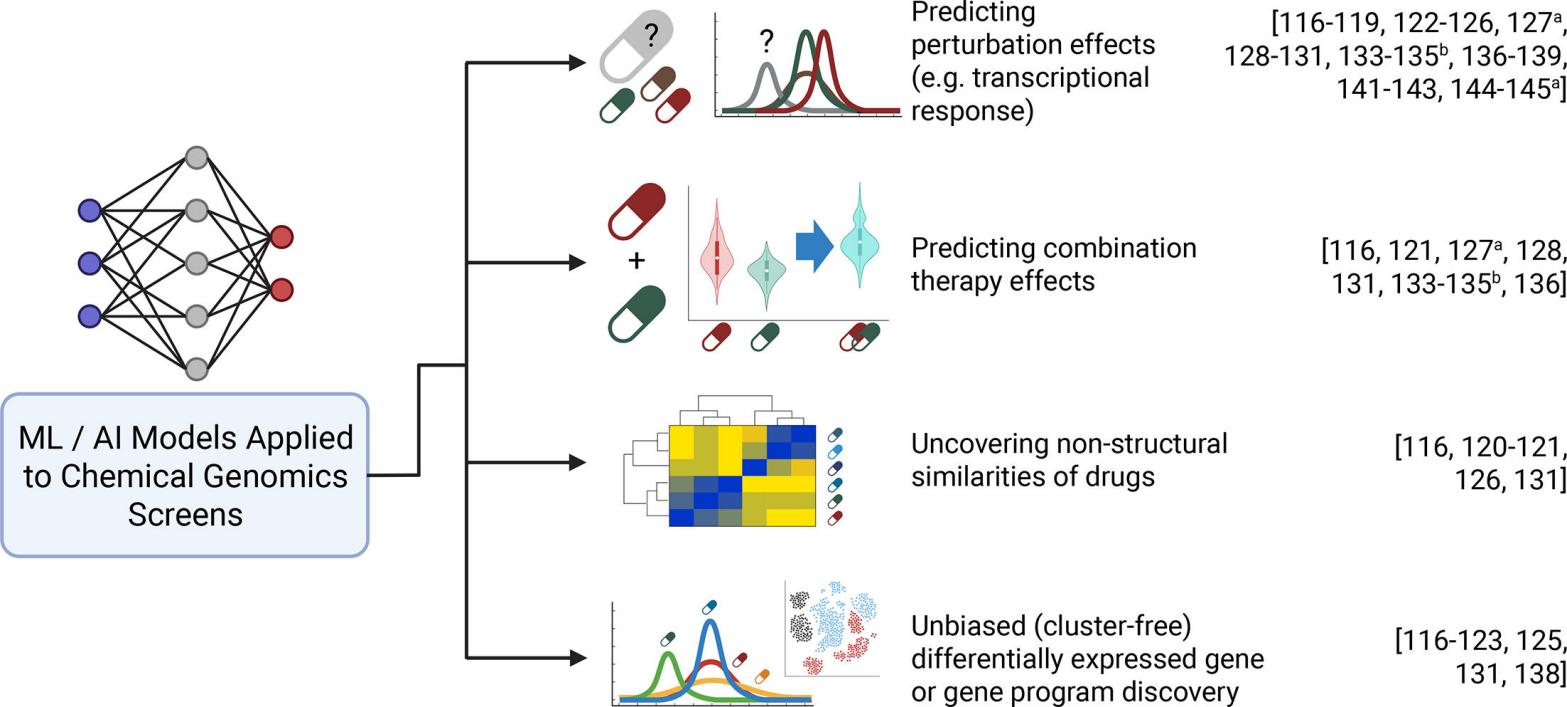
Overview of downstream analysis tasks performed by computational methods trained on chemical genomics screens. Left, graphical representation and description of the task; right, citation number of methods that can perform the task. ^a^Task achieved by method trained on genetic but not chemical perturbations as of publication. ^b^Task possible through *in silico* gene regulatory network (GRN) perturbation but not explicitly tested by the method as of publication.

Advances in machine learning (ML) methods applied to scRNA-seq to learn and detect perturbational response patterns have accompanied the generation of single-cell perturbation datasets [116]. A key challenge is that perturbation responses are typically measured on different cells before vs. after treatment, yielding unpaired single-cell data distributions. Another crucial objective in perturbation modeling is out-of-distribution prediction, or predicting treatment effects from unseen conditions, given that it is infeasible to explore the full combinatorial space of drug perturbations. A model must first learn perturbation-induced transcriptional effects and subsequently, the model can predict transcriptional effects from counterfactual conditions, including untested doses, treatment timings, responses from different cell types, or drug combinations. Further complexities include disentangling perturbation-specific effects from confounding variations, and modeling drug–drug interactions across diverse contexts may be challenging. In this section, we discuss recent exciting advances in ML methods that can be used to uncover biology from single-cell chemical genomics screens, as well as forward-looking directions involving pre-trained single-cell foundation models and single-cell causal discovery methods (Figure 3).

Modern representation learning methods have emerged as the computational backbone for single-cell chemical genomics analysis, leveraging the flexibility of variational autoencoders (VAEs) and hierarchical Bayesian modeling [117,118]. These approaches excel at reconstructing expression data while learning latent variables relating to cell states or modules of gene expression, providing denoising capabilities through probabilistic modeling [119–126]. Specifically, representation learning models feature generative processes that reconstruct expression profiles of seen conditions using approximated probability distributions of latent factors to summarize cell state (encoders) and mappings from latent factor reduced dimensions to expression space (decoders). Notably, the decoding process can be leveraged to predict transcriptional treatment effects from counterfactual conditions not represented in the original training set, depending on model design [117].

In just the past 2 years, an assortment of representational learning models has aimed to predict treatment effects on the single-cell transcriptome as a central task. The Compositional Perturbation Autoencoder (CPA) encodes conditions as independent covariates using adversarial loss to predict counterfactual expression profiles using linear combinations of latent factors and covariates [127]. This approach builds on concepts from the authors’ prior scGen model that combines VAEs with latent space vector arithmetic to model how a perturbation shifts gene expression in unseen cell types [128] as well as their trVAE model that uses maximum mean discrepancy regularization to force cross-condition similarities and enable out-of-distribution predictions [129]. scVIDR additionally uses log-linear interpolation to predict cell type-specific, dose-dependent gene expression changes across unseen doses [130]. While these VAEs learn and encode perturbational effects as latent factors assumed to be consistent across cell states, MrVI (Multi-resolution Variational Inference) more generally models heterogeneous transcriptional response across nested multi-condition experiments, which can then be used for multiple downstream analyses, including computing transcriptomic similarities between seen and unseen conditions and predicting counterfactual differential expression for a given cell state [131]. Relatedly, DRVI employs additive decoders along with pooling functions to learn disentangled latent representations, in this case corresponding to distinct drug or gene perturbation transcriptional effects [132]. The sVAE+ and FCR methods aim to learn causal representations of chemical or genetic perturbations on single-cell transcriptomics readouts using sparse mechanism shift modeling and factorized causal representation learning, respectively [133,134]. Notably, FCR aims to distinguish treatment and covariate (e.g. cell line) specific effects, as well as interaction effects between treatments and other covariates, which may be useful for disease-specific prioritization of perturbation targets.

The recent scCADE method also tackles decoupling cellular context from perturbation responses using contrastive learning and attention mechanisms towards predicting unseen perturbation effects in seen cellular contexts [135]. Another recent method, LEMUR, avoids discrete categorization of cell states across experiment conditions and instead relies on matrix factorization using pre-specified conditions as covariates to map cell phenotypes to a continuous latent space and predict cell-state specific differential expression between conditions, although this approach may not reflect non-linear treatment effects [136]. OntoVAE incorporates biological ontologies into latent space representations, which allow for direct interpretation of simulated drug or gene transcriptional effects, although the model may not distinguish causal from direct relationships within the biological ontology space [137]. Conversely, scCAPE employs adversarial learning to distinguish perturbation effects on the single-cell transcriptome from intrinsic or extrinsic cell state variation, the latter being captured in scCAPE’s latent space. However, scCAPE was originally designed for genetic perturbations (i.e. Perturb-seq training data), and perturbation inference is conducted at a factor and not a gene-specific level [138].

Modeling perturbations can be formulated as identifying a mapping between two distinct, unpaired single-cell expression datasets. As such, optimal transport has been proposed as a framework to infer or predict treatment effects. CINEMA-OT proposes optimal transport within a causal framework to isolate confounding variation and infer causal treatment effects at the single-cell level via counterfactual cell pairs, which can also be used to predict combinatorially perturbed phenotypes [139]. CellOT and the more high-dimensionally efficient W1-OT framework operate within a non-causal context to predict perturbation effects in single-cell expression data [140,141].

Finally, the BATCHIE framework utilizes active learning and flexible Bayesian modeling to enable adaptive experimental design for efficient and informative combinatorial drug screens. While not a fixed model, BATCHIE explicitly encourages iterative and rational drug screening to explore the combinatorial space, which we predict will be a crucial next direction in the field of chemical genomic screening [142]. In turn, rational generation of combinatorial perturbation data will further improve the performance of the aforementioned methods in modeling the full combinatorial perturbation space [127,132,138,139]. In summary, ML methods have shown great performance and promise in dissecting biology from large-scale experimental single-cell datasets.

Beyond predicting phenotypic responses, computational methods and causal discovery techniques are harnessing perturbation screens to reverse-engineer gene regulatory networks (GRNs). GRNs capture co-regulatory dynamics, specifically transcription factor (TF) and target gene interactions, and yield insight into the molecular underpinnings of disease. A well-constructed GRN could simulate dose-dependent or combinatorial effects of chemical or genetic perturbations by propagating perturbation effects across connected nodes (e.g. *in silico* knockdown, knockout, and/or overexpression of multiple TFs or kinases). Robust GRN inference from scRNA-seq has been a long-standing goal in the field of bioinformatics and systems biology [143]. Unfortunately, directly inferring GRNs from experimental scRNA-seq alone, for example, through simplistic covariance analysis or specialized tools like SCENIC [144], often fails due to the static nature of single-timepoint gene expression and other technical limitations like noise and sparsity. An active area of interdisciplinary ML research applies novel causal discovery methods to better infer GRNs from genetic perturbation scRNA-seq [145,146]. Genetic perturbation scRNA-seq (e.g. Perturb-seq) is the gold-standard modality to study gene–gene relationships for GRN inference, although noise and sparsity inherent to genetic screens continue to impede robust GRN inference. Nonetheless, we anticipate future causal approaches will increasingly use deeper and wider chemical screens as training datasets instead.

Contemporary deep generative methods like PDGrapher, graphVCI, and TFdisc train on chemical, genetic, and/or no perturbation (wildtype, control) scRNA-seq datasets to predict transcriptional effects using already-constructed or inferred GRNs. PDGrapher predicts combination chemical perturbations necessary to reverse a given disease’s effects given ground-truth GRNs, while TFdisc simulates TF perturbation by learning inter-regulation between TFs and target genes from wildtype scRNA-seq using random forests. graphVCI builds upon a previous variational causal inference framework VCI and trains on either chemical or genetic perturbation data to refine given ground-truth GRNs and predict cell-specific counterfactual transcriptional effects from unseen perturbations [147–150]. Key challenges remain in inferring GRNs, such as deconstructing cyclic relationships between feedback-looping genes in favor of directed acyclic graphs, but we are excited about the utility of these methods and their emerging application to analyzing chemical genomics screens.

Finally, in the advent of popular large language models enabled by generative pre-trained transformers (GPTs) such as ChatGPT, LLaMA, and DALL-E, we anticipate self-supervised foundation models (FMs) will play a large role in future efforts to predict drug-induced molecular phenotypes. FMs can be pre-trained on diverse, large-scale single-cell datasets to encode biology or other useful representations and are an attractive option in order to subsequently perform various downstream tasks with minimal-to-no refinement. Several single-cell FMs have been developed with diverse performance across common analysis tasks within single-cell genomics pipelines, with impressive performance already noted in automated cell type annotation and strong headways in predicting gene expression changes following genetic perturbations [151]. Notably, scGPT can predict unseen genetic perturbation responses and infer genetic networks, scFoundation features both drug and genetic perturbation response predictions as downstream tasks, and other single-cell FMs may indirectly yield insight into drug response at the expression level across tissue types or species [152–156]. While FMs already show promise in single-cell data analysis, their application to chemical genomic screens to perform downstream analyses such as predicting dose-dependent or combinatorial drug effects is yet to be tested, and it remains unclear whether they currently outperform the aforementioned methods.

We anticipate a new era of transfer learning in chemical genomics where pre-trained models can be adapted to predict any chemical or genetic perturbation combination in diverse contexts. Such *in silico* predictions could greatly accelerate virtual screening of drug effects, hypothesis generation for combination therapies, and discovery of causal regulatory mechanisms through providing a flexible backbone that had already learned the language of cells [157]. A central challenge is the lack of a large corpus of single-cell chemical genomics datasets necessary for pre-training existing and future model architectures and refining single-cell FM pre-training strategies. While biopharma industry-sponsored initiatives have made headway into generating large-scale, single-cell drug perturbation datasets [75], we predict comprehensive training datasets in single-cell genomics will remain elusive in the near term. Future efforts in integrating causal structures into attention-based FMs would be exciting directions to ensure interpretability.

## Outlook and future perspectives

The integration of single-cell molecular profiling into chemical genomics and drug discovery has demonstrated its potential to transform our understanding of drug mechanisms, polypharmacology, and therapeutic efficacy. Looking ahead, we expect that advancements in single-cell methodologies will greatly refine our ability to perform drug target identification, characterize correlates (biomarkers) of therapeutic response, and predict resistance mechanisms a priori.

The resolution provided by single-cell genomics has provided key insights into the cell type(s) most likely to be affected in a disease and make predictions regarding how cellular heterogeneity and shifts in cell state(s) co-vary with disease phenotypes and an individual’s response to therapy [15,17,62,158–163]. This ability to refine target identification can help ensure that therapeutic interventions are directed at the most relevant cellular subpopulations. In the context of the chemical genetics paradigm, single-cell chemical genomics profiling has more deeply defined the consequence of drug exposure capturing molecular and cellular determinants of efficacy and resistance [62,114,164,165], which can ultimately guide the design of more effective combination therapies.

In the context of cancer, various screenable modeling approaches have been developed to facilitate the high-throughput screening of drug response at single-cell resolution. Established cell lines using multiplexed sequencing methods have provided insights into how genetic background influences drug response [5,102] and are rapidly scalable and multiplexable [75]. Patient-derived models have been optimized to maintain tumor heterogeneity [100,166], which can improve the clinical relevance of preclinical findings. Organoid models have allowed for the study of drug responses in three-dimensional environments that mimic *in vivo* conditions [167], while explants have preserved native tissue architecture and cell–cell interactions [165,168]. However, a systematic analysis to identify minimal screenable systems for a given molecular phenotype (i.e. retain key disease and therapeutic response features while maintaining multiplexing ability) is still lacking. Looking ahead, extending single-cell chemical genomics to more physiologically relevant model systems will be essential. Organoids [169], patient-derived explants [165], and microphysiological ‘organ-on-chip’ platforms [170] can better capture native 3D architecture, microenvironment interactions, and gradients in drug exposure that shape therapeutic response. Key opportunities lie in adapting these complex systems to high-throughput profiling and perturbation screens and in integrating spatially resolved readouts that map transcriptional drug responses back to defined structural features. Lastly, evolution under therapy due to genetic variation [171] or cellular plasticity [172] has also been identified as a manner by which tumors evade the effects of therapy. Incorporation of single-cell lineage tracing [104,173] will be instrumental in determining how cell populations evolve in response to drug exposure and has already highlighted how clonal expansions contribute to therapeutic resistance to chemotherapy [174].

Future promising directions in single-cell chemical genomics include the integration of spatial transcriptomics [175–179] and spatially barcoded single-cell transcriptomics approaches [180,181] to capture tissue-specific drug responses to drugs and leverage optical molecular screens [182–184] to define their regulation. While artificial intelligence (AI) and ML hold promise for advancing drug discovery, a major challenge lies in the need for comprehensive, large-scale, high-quality single-cell perturbation datasets to train these models effectively, a challenge that is excitingly beginning to be met by the release of the Tahoe-100M datasets [75].

The next decade holds immense promise for single-cell molecular profiling in drug discovery. By integrating high-throughput single-cell screening with computational innovations and multi-modal approaches, researchers will unlock deeper insights into drug mechanisms of action, accelerate the identification of effective therapies, and refine personalized treatment strategies. While challenges remain in scalability, data interpretation, and clinical translation, continued interdisciplinary advancements will solidify single-cell chemical genomics as a cornerstone of modern drug development.

## Open Access

Open access for this article was enabled by the participation of Columbia University in an all-inclusive Read & Publish agreement with Portland Press and the Biochemical Society.

## Abbreviations

ATAC-seq: assay for transposase accessible chromatin
CMap: Connectivity Map
FMs: foundation models
GPTs: generative pre-trained transformers
GRN: gene regulatory network
ML: machine learning
NGS: next-generation sequencing
PDD: phenotypic drug discovery
STAMPs: single-cell transcriptomes attached to microparticles
TDD: target-based drug discovery
TF: transcription factor
VAE: variational autoencoders
sgRNA: single guide RNA
